# Surface modifications of titanium dental implants: optimizing antibacterial activity and osteoimmunomodulation

**DOI:** 10.3389/fbioe.2026.1795260

**Published:** 2026-05-19

**Authors:** Samar Shurbaji, Ahmed Malki, Hassaan Anwer Rathore

**Affiliations:** 1 College of Dental Medicine, QU Health, Qatar University, Doha, Qatar; 2 Biomedical Science Department, College of Health Sciences, QU Health, Qatar University, Doha, Qatar; 3 Department of Pharmaceutical Sciences, College of Pharmacy, QU Health, Qatar University, Doha, Qatar

**Keywords:** antibacterial, osteoimmunology and osseointegration, osteoimmunomodulation, peri-implantitis, surface modification, Titanium dental implants

## Abstract

Titanium (Ti) and its alloys are the gold standard for dental implants and orthopedics generally. This is mainly attributed to their exceptional mechanical properties, corrosion resistance and high biocompatibility. Regardless of their clinical success, Ti suffers from certain drawbacks including the slow osseointegration due to the bio-inertness of Ti, additionally, their surface is susceptible to bacterial adhesion leading to biofilm formation and then causing condition like peri-implantits and implant failure. For that, achieving a long-term successful implant requires addressing a dual challenge, mainly to achieve high tissue integration with limiting bacterial colonization. This review paper compiles the innovative strategies in Ti surface engineering for the past 15 years. Mainly, focusing on developing multifunctional implants that have high osseointegration capacity with antimicrobial properties. The review progress from discussing the conventional modification methods such as etching and grit blasting, to advanced physiochemical approaches, including nanoscale modification, biomimetic functionalization and stimuli responsive technologies. This review highlights strategies to integrate functional components by modifying surface topography, charge and wettability. For example, approaches like topographical modifications, inorganic and organic modifications are discussed. A considerable focus is addressed to the role of osteoimmunology, mainly on how surface modifications can modulate the host immune response by promoting the polarization of the anti-inflammatory M2 macrophages with dominance over the M1 type which induce inflammation. Further thing this review highlights is the advanced smart surface designs that respond to internal or external stimuli for on request release of drugs or antimicrobial agent activation. Finally, translational challenges are discussed including the need for long term clinical data, enhanced mechanical stability, and tracking of regulatory concerns regarding the nano-toxicity and ion release. Future directions are directed towards designing immune instructive and dynamically responsive implants that move beyond passive biocompatibility but toward active biological integration.

## Graphical Abstract

**Figure:**
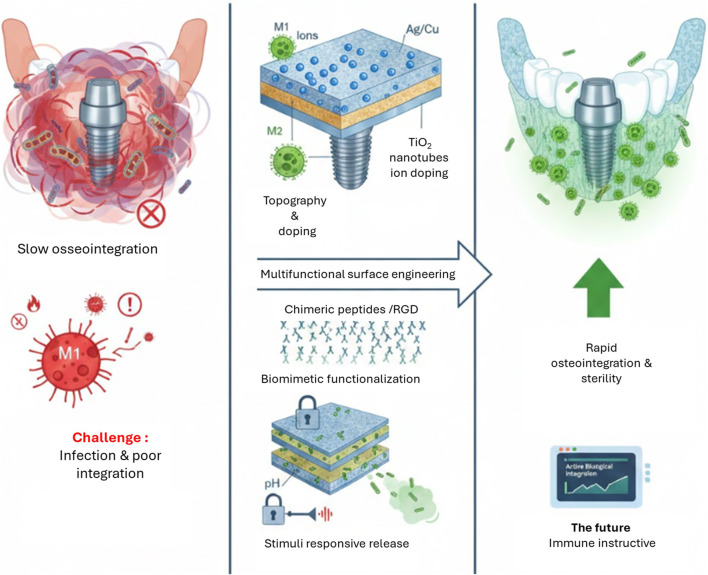

## 1 Introduction

The need to replace a missing tooth has been ongoing since ancient times, for example, the ancient Egyptians used gold wires, the Mayan’s (700 AD) used seashells, actual human teeth were used in the 15th–18th century. Ivory and wood in medieval times were used. All these techniques showed some success but were not completely effective (Abraham, 2014). The modern history of dental implantology was transformed in the 1952 where a Swedish orthopedic surgeon Per-Ingvar Brånemark, accidentally discovered during his intravital microscopy studies of bone marrow in rabbit. Brånemark noticed that Titanium chambers used in the experiments have completely fused with the bone tissue. He named this phenomenon” osseointegration” which means a direct structural and functional link between the bone and implant surface. This discovery paved the way for the first successful Titanium implant in a human patient in 1965 (Agarwal et al., 2024).

Titanium (Ti) and its alloys are generally the gold standard material for dental implants and orthopedics worldwide (Souza et al., 2019; Wang et al., 2023; Kang et al., 2025; Zhang et al., 2025a). This is mainly attributed to its exceptional characteristics including high strength to weight ratio, outstanding mechanical properties, corrosion resistance and high biocompatibility. Titanium-based dental implants have a notably high success rate which is 87.8% over a period of 36 years (Souza et al., 2019). Although Ti has widespread clinical success, it has some critical drawbacks, one of these is the bioinert nature of Ti which limits its osteogenic and osteoinductive abilities (Han et al., 2023). In addition to these drawbacks, the success and survival of such implants are highly affected in patients with systemic health issues. Uncontrolled cases of diabetes mellitus, a habit of heavy smoking, and the intake of antiresorptive medications (such as bisphosphonates) have been identified to negatively affect the biological process of healing and result in peri-implantitis. In medically compromised patients, the natural microenvironment is often associated with poor vascularization and an unbalanced inflammatory response, resulting in poor bone formation. There is, therefore, a pressing clinical need for the optimization of Ti implant surfaces that can proactively address these systemic disadvantages and provide predictable osseointegration even in a biologically challenging microenvironment (Tuikampee et al., 2024).

While Ti remains the gold standard, Zirconium based materials (zirconia/Zr) and Ti- Zirconium alloys have gained significant popularity (Cionca et al., 2000; Ciszyński et al., 2024). Zirconia implants offer superior esthetics due to their tooth like color and exhibit low bacterial adhesion affinity, which may reduce the risk of peri-implantitis (Mohseni et al., 2023). However, they are prone to low temperature degradation and have a higher risk of mechanical fracture compared to Ti. For that Ti/Zr alloys (such as RoXolid – 85% Ti/15% Zr) have been developed offering significantly higher tensile and fatigue strength than pure Ti. These alloys allow for the use of smaller diameter implants without compromising the mechanical integrity, nevertheless they still carry the risk of metal ion release associated with Ti based materials (Grandin et al., 2012). Beyond metallic and ceramic options, high performance polymers such as polyether ether ketone (PEEK) have emerged as popular alternative (Panayotov et al., 2016). Unlike Ti, PEEK has low modulus of elasticity which closely resembles the human cortical bone, thereby reducing the stress shielding and bone resorption (Najeeb et al., 2016). Additionally, PEEK is chemically inert, non-allergenic and offers excellent radiolucency allowing artifact free clinical imaging. However, PEEK is inferior to Ti in terms of cell adhesion and ossessiointergation. For that Ti is still superior material in terms of bioactivity (Senra et al., 2023).

The implant stability is defined by the success of osseointegration which in case of titanium is slow as it takes from three to 6 months. Additionally, pristine Ti does not have any antimicrobial properties and is highly vulnerable to bacterial adhesion and biofilm formation (Kang et al., 2025; Hu et al., 2022; Sánchez-Bodón et al., 2021; Sobolev et al., 2019). Due to these drawbacks, many complications can occur such as delayed healing, implant instability and failure and the need for subsequent surgeries. Moreover, the biofilm formation can lead to further complications and inflammatory destruction such as peri-implantitis. Consequently, to achieve a long-term successful implant, a dual challenge should be addressed: inadequate tissue integration, and the susceptibility to microbial contamination (Kang et al., 2025; Selvamani et al., 2022; Sun et al., 2019; Li L. et al., 2025).

For that, the development of multifunctional Ti implants that have a good biocompatibility, high osseointegration and antimicrobial properties is highly needed. The most important part when designing an implant is to ensure that the surface promotes bone integration instantaneously and prevent bacterial colonization (Li et al., 2022; Li et al., 2023; Chuang et al., 2025). Achieving this involves “winning the race for the surface,” in this case, rapid cellular adhesion should be achieved before bacterial adhesion and proliferation (Selvamani et al., 2022; Piñera-Avellaneda et al., 2023). This dual role must be achieved while not harming the nearby tissue or disturbing the osteoimmunological balance required for healing (Li et al., 2023; Chuang et al., 2025; Piñera-Avellaneda et al., 2023). To achieve that, different strategies are involved such as micro and nano scale modifications, surface functionalization using proteins, peptides, bioactive ceramics and antimicrobial agents (Souza et al., 2019).

Despite the significant advancement in surface modification strategies, translating them to clinical practice remains challenging (Zhang et al., 2025a; Antonoglou et al., 2025). As being associated with long term complications related to aseptic loosing, infection and mechanical issues such as wear, material fatigue and excessive mechanical loading with stress shielding caused by modulus mismatch. Such factors affect the implant’s survival rate which leads to late-stage implant failure. To resolve this, material modification should overcome many issues including enhancing the mechanical integrity to achieve long-term stability. Additionally, to improve the drug release approach (concentration) to avoid antibiotic resistance and cytotoxicity (Chuang et al., 2025; Hasani-Sadrabadi et al., 2021; Buga et al., 2021; Petersen, 2014; Damiati et al., 2018).

While modifying Ti is the primary focus, alternative materials offer important benchmarks, for example, zirconia has been introduced to implant dentistry due to its high biological and mechanical performance (Antonoglou et al., 2025; Petersen, 2014). Other materials like polymer matrix composites (PMCs) are being investigated as alternative potential solutions. The aim is to benchmark Ti against other alternative solutions (Petersen, 2014).

**FIGURE 1:**
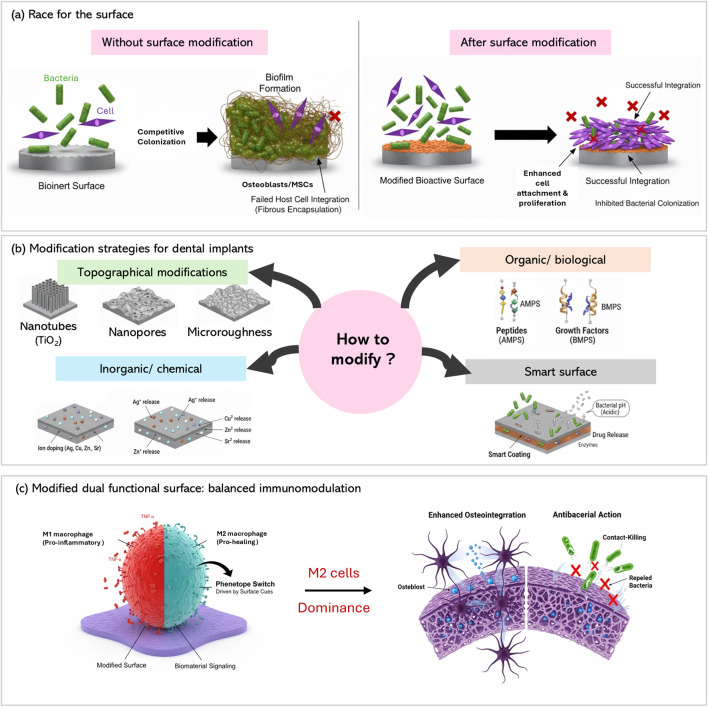
The evolution of Ti surface modifications from bio-inert to immune modulating dual functional implants. **(a)** Comparison between modified and unmodified Ti surfaces, and competitive colonization between the host cells and bacteria. **(b)** Different modification methods to establish a surface with good osseointgrative properties while being bactericidal. **(c)** Dual surface functionalization leads to M2 dominance and enhanced osseointegration.

**FIGURE 2:**
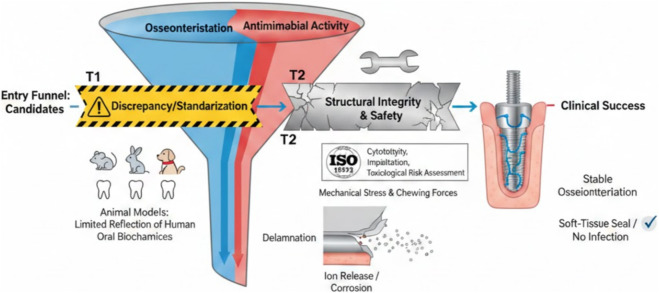
This figure shows the complexity to achieve a dental implant material that has dual functionality and can be used clinically. Such material should meet both bone integration (blue path) and microbial control (red path). Certain considerations should be put into account including standardization, animal use, general safety and toxicity that might be caused due to release and corrosion. Controlling all these factors will generally lead to implant stability and success.

This mini review aims to compile and analyze the innovative strategies in Ti surface engineering for the past 15 years (2010–2025). The progression from conventional modification methods such as grit blasting and etching to advanced physicochemical approaches such as nanoscale modification, biomimetic functionalization, and stimuli responsive nanotechnologies. With emphasis on using those recent developments to integrate variety of functional components to optimize implant performance. Figure 1 summarizes the overall paper concept. Figure 2 represents the complexity of achieving an optimum implant surface with dual functionality.

## 2 Core conflict: understanding the dual mechanism

The successful functioning of a dental implant depends highly on a dual mechanism: achieving sufficient tissue integration while limiting bacterial colonization. This dual functionality creates a conflict in biomaterial design, mostly referred as a challenge of increasing the therapeutic window (Wang et al., 2023; Parizi et al., 2022). This challenge is critical as microbial infection and insufficient tissue integration are the main two causes of implant instability and failure (Wang et al., 2023; Safaei et al., 2024).

### 2.1 Pathogenesis of peri-implantitis and bacterial adhesion

Peri-implantitis is a plaque associated pathological condition that occurs in tissues surrounding the dental implant, this condition is mainly characterized by inflammation and continuing loss of supporting bone (Sousa et al., 2022). It is the most common complication in dental implantology with prevalence rate ranging from 7% in healthy populations to 38.4% in subjects with 10 years’ functional implants. (Parizi et al., 2022).

**FIGURE 3:**
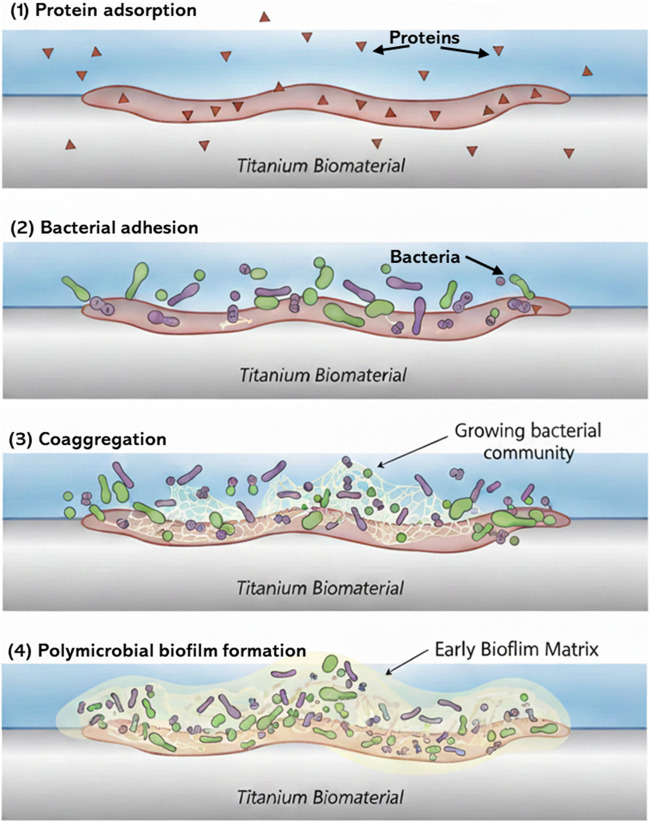
stages of polymicrobial biofilm development on a Ti biomaterial. **(1)** Presents protein adsorption on the surface, followed by **(2)** bacterial adhesion to the protein layer **(3)** coaggregation of different bacterial species leading to the formation of polymicrobial biofilm **(4)**. *Adapted from* (Gonzalez et al., 2020) *with modifications*.

The pathogenesis of peri-implantitis is mainly driven by a major microbiological component, which involves destruction of both hard and soft tissues at the implant site due to the inflammatory process caused by bacterial contamination (Antonoglou et al., 2025). Following the implant placement, the race for the surface begins. This process begins immediately (within 30 min) after surgery as the plasma proteins and salivary components coat the implant surface, thus allowing oral microorganisms to adhere to the surface and initiating microbial accumulation (Diez-Escudero et al., 2022). This bacterial adhesion leads to the formation of peri-prosthetic biofilm layer, once formed, is difficult to eradicate by conventional antimicrobial treatments. Usually, oral biofilms are polymicrobial involving fungi such as *Candida albicans* beside bacteria such as *Streptococcus mutans* and *Streptococcus.aureus*, such microbial combination can synergize the virulence of the plaque biofilm (Gonzalez et al., 2020). Figure 3 illustrates the steps in which bacterial adhesion and biofilm formation are formed.

### 2.2 Surface modification impact on host-microbiome interaction

The impact of surface modification on host microbiome interaction has been greatly studied, yielding complex and diverse results across different approaches. This section discusses the impact of different surface modifications on host bacterial interactions.

#### 2.2.1 The influence of surface topography and wettability

Physical surface properties significantly dictate the outcome of host microbiome race, nonetheless research found that it’s much more complex to be put in a simple topographical rule As Wu et al. suggested that simple roughness measurements are not enough for the prediction of the success of titanium implants, instead the key factor is the scale of the surface features. Particularly, surfaces the feature roughly the size of bacteria dramatically increases infection, while those with larger features successfully promote bone growth and minimize bacterial colonization (Wu et al., 2011). Further study suggests that rougher Ti surfaces favored osteoblastic viability and differentiation, whereas bacterial viability and metabolic activity were broadly similar across different surface treatments (Rodriguez-González et al., 2023). More recently, Tardelli et al. reported in their systematic review that surface treatment that reduce areas like potintiostat anodization and lasers, favors bacterial adhesion, such surfaces lead to significant biofilm interference more than surface roughness alone. Also, they suggested that bacteria prefer surfaces with similar hydrophobicity as hydrophobic bacteria prefers hydrophilic surfaces and it adhere to it by forming hydrogen bond. These findings emphasize the role of wettability in bacterial surface adhesion (Tardelli et al., 2023), as Choi et al. reported that increasing surface hydrophilicity from 38° to 72° resulted in significantly higher biofilm formation and adhesion compared to the control surfaces (Soares, 2024). Additional study reported the role of residual stresses that form after grit blasting on both smooth and rough Ti surfaces. They found that compressive residual stress on both smooth and rough surfaces improves the hydrophilicity and increases the surface energy. Such improvement led to enhancement in osteoblastic proliferation and did not increase the bacterial colonization (Pereira et al., 2024).

#### 2.2.2 The role of surface charge, potential and photocatalysis

Altering the surface electrostatic conditions and chemical potential offers approaches that change the surface properties to reduce bacterial adhesion without relying on surfaces/coatings that release chemical agents. In their study Shen et al. reported that positively charged hydrophobic surface created via (3-aminopropyl) triethoxysilane (APTES) modification showed superior adhesive resistance against different bacteria. Their outcomes suggested that the APTES modification eliminated implant associated infection in rat model, as histological findings showed lack of inflammation in APTES implants compared to untreated Ti however this study is limited to the short study duration (Shen et al., 2020). Furthermore, Kreve and Reis concluded in their systematic review that electrostatic perturbation on Ti surface causes a decline in bacterial growth/development of antimicrobial surface. However, due to many discrepancies in the applied factors in the included studies, it was impossible to clearly state that the electrostatic conditions alone contribute to such antimicrobial surface development (Kreve and Cândido Dos Reis, 2021). Additional study reported by Pan et al. where they investigated photo activated Ti surfaces, they found that the UV exposed surfaces have time dependent bactericidal activity, thus resulting in significant reduction in bacterial colony forming units for *P aeruginosa* and *S. aureus* after 24- minutes UV irradiation (Pan et al., 2021). The clinical relevance of this *in-vitro* study is limited since the UV exposure was not controlled.

#### 2.2.3 Antimicrobial peptide and chimeric peptide modifications

Engineered biological molecules not only eliminate bacteria but also help in recruiting host cells. As Zhang et al. reported the effect of incorporating a GL13K releasing hydrogel in Ti implant surface, they concluded that this incorporation promoted the differentiation of osteoblasts *in vitro* (Zhang et al., 2023). Further study reported the modification of Ti surface using chimeric peptide (TBP-1-RGDS-hBD3-3), such modification resulted in antimicrobial activity against initial biofilm forming bacteria, however, this *in-vitro* approach lack the host immune complexity (Zhang et al., 2018a).

#### 2.2.4 Drug and metal ion incorporation

Incorporating therapeutic agents provide direct antimicrobial action combined with osseointegration promotion. The incorporation of antibiotics was reported by Sharma et al. in their study as they designed gentamicin-loaded silk fibroin nanoparticles (Ti-GNp). This produced formulation successfully achieved a sustained drug release *in vitro* and showed a reduced adhesion of *s.aureus* compared to uncoated Ti surface. Gentamicin did not cause any harmful effect on the cellular adhesion, proliferation and general function. While the overall coating enhanced all these parameters compared to untreated Ti surfaces, this is a promising approach however it lacks the host complexity (Sharma et al., 2016). More recent report by Standert et al. As in their study they prepared amphora shaped porous Ti implant with gentamicin loading, this formulation effectively prevented infection in rat model alongside possessing a good cyto and biocompatibility. This study provided a useful proof of concept however limited to species differences and short follow up period (Ständert et al., 2021). Further study where antibiotic mix was used, was studied by Alvisi et al. in their *in vitro* study they investigated the effect of combining antibiotic mixtures on rough surfaces, this combination showed a bactericidal efficacy almost twice that with rough surfaces alone. The translation of this approach is limited due to the risk of antimicrobial resistance and dosing challenges (Alovisi et al., 2022). The incorporation of metals like manganese is believed to reduce the bacterial adhesion and induce osteogenesis. In their study Yu et al. showed that manganese (Mn) incorporated Ti surfaces with stable Mn release. This formulation showed a decline in gram negative bacteria viability with significant enhancement in osteogenesis–related gene expression. This study is limited due to the absence of host immune complexity as well as mechanical loading factors (Yu L. et al., 2017). Additional study by Shen et al. reported Ti surface modified with hydrophilic Zn- incorporated nanowires. Their formulation demonstrated a dual effect *in vivo* as it enhanced osseointegration and suppressed inflammation by inhibiting multiple gram positive and negative species. This approach is promising but limited to the absence of long term human data (Shen et al., 2024). Other antimicrobial agents were reported as Celesti et al. reported the functionalization of Ti surfaces with quaternary ammonium salts and oleic acid, such formulation showed exceptional results in terms of bacterial adhesion inhibition against both *E. coli* and *S aureus* (Shen et al., 2024).

**FIGURE 4:**
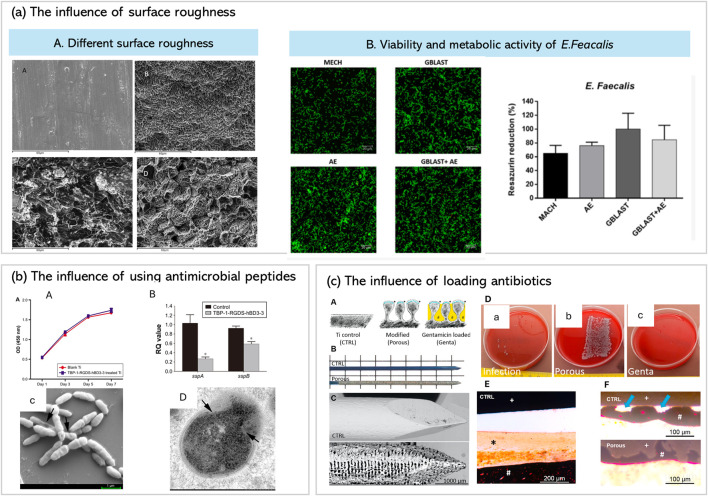
Represents the effect of different surface modifications on bacterial growth and colonization. **(a)** represents the effect of surface roughness after 4 types of surface modifications (machined “MACH), (acid etched “AE”), (grit-blasted “GBLAST”) and (grit blasted with acid etching “GBLAST+AE”) on bacterial colonization **(b)** represents the influence of using antimicrobial peptides. in which chimeric peptides combining Ti-binding, RGD, and hBD3 motifs inhibit streptococcal colonization and biofilm development, with evidence of membrane disruption and reduced gene expression (sspA/sspB). **(c)** Antibiotic loading into amphora-shaped laser-structured pores: gentamicin-loaded porous titanium surfaces demonstrate enhanced antibacterial efficacy and osseointegration, with reduced infection signs *in vitro* and *in vivo*. *Adapted from* (Rodriguez-González et al., 2023; Wu et al., 2011), (Shen et al., 2024), (Ständert et al., 2021), (Zhang et al., 2018b) *with permissions*.

These collective findings indicate that successful surface modification strategies often involve modifications that perform multiple functions (antibacterial and cell growth promotion) such strategies involve incorporating antimicrobial agents, tailoring the surface characteristics and physiochemical properties. Figure 4 summarizes different research outcomes regarding the impact of different surface modifications on bacterial adhesion and growth.

Taken together, these approaches highlight a trade-off: while nanoscale roughness and hydrophilicity promote osteoblast adhesion, they may also favor bacterial colonization. Conversely, electrostatic and photocatalytic modifications reduce bacterial adhesion but face translational limitations. The controversy remains over whether surface roughness or wettability is the dominant factor in clinical outcomes, as systematic reviews report conflicting evidence.

### 2.3 Osteoimmunology: macrophage function, cytotoxicity/bactericidal balance, and the road to osseointegration

To achieve a successful Ti dental implant, the design should have a delicate balance between effective antibacterial activity and osseointegration promotion. To get such outcome, understanding and modulating the osteoimmune microenvironment is heavily needed (Chen et al., 2020; Huang et al., 2018).

#### 2.3.1 The role of osteoimmunology and macrophage response in osseointegration

The response of the host immune system to the implanted biomaterial is a critical factor affecting the fate of bone-implant integration (Buga et al., 2021; Petersen, 2014). The concept of osteoimmunomodulation suggests that implant design must actively regulate the immune microenvironment to favor bone healing, shifting from the traditional approach of creating bio-inert materials (Zhao et al., 2021; Zhang et al., 2019; Qiao et al., 2020).

Once the implant is inserted after the surgery, multiple steps and immune interactions happen. Neutrophils the cells the come first to clear the surgical site, followed by monocytes. Monocytes will then differentiate to macrophages; macrophages act as key mediators in the early immune response to the foreign implantable material. Typically, macrophages arrive at the surgical site within hours of surgery, the macrophages response to the implant decide the subsequent processes of tissue healing and remodeling. As macrophages exhibit high plasticity and differentiate in spectrum, mainly classified as two extremes: M1, pro inflammatory and M2, anti-inflammatory (Zhu et al., 2021; Baseri et al., 2020). The transition from M1 to M2 macrophage polarization is more than just an indicator of healing; it is a driving force behind osteogenesis. M2 macrophages release pro-osteogenic cytokines, such as Bone Morphogenetic Protein-2 (BMP-2) and TGF-β, which function as paracrine factors to induce adjacent Mesenchymal Stem Cells (MSCs). These factors initiate the Wnt/β-catenin signaling pathway, resulting in the translocation of β-catenin into the nucleus, where it binds with T-cell factor/lymphoid enhancer factor (TCF/LEF) transcription factors. This complex eventually activates the transcription of osteogenic genes such as Runx2 and Osterix, thus establishing a link between the immune response and the physical process of bone formation (Guder et al., 2020).

**FIGURE 5:**
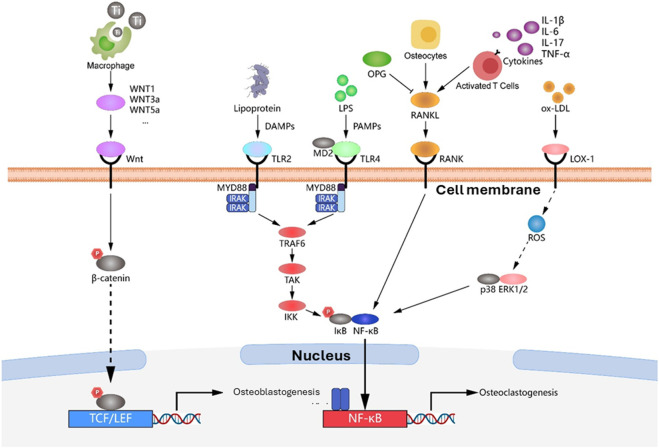
Molecular signaling pathways regulating the balance between osteogenesis and osteoclastogenesis at the implant interface. This diagram illustrates the signaling mechanisms determining bone fate. The left side depicts the M2 macrophage-derived Wnt ligand activating the Wnt/β-catenin pathway; this leads to the nuclear translocation of β-catenin and its binding with T-cell factor/lymphoid enhancer factor (TCF/LEF) transcription factors to induce osteogenesis. Conversely, the right side represents the inflammatory response where bacterial Lipopolysaccharides (LPS) and lipoproteins act on Toll-like receptor 4 (TLR4) and Toll-like receptor 2 (TLR2), respectively, while cytokines activate the Receptor Activator of Nuclear Factor kappa-B (RANK). This triggers Nuclear Factor-kappa B (NF-κB) activation and Reactive Oxygen Species (ROS) generation, leading to the expression of osteoclastogenic genes, bone resorption, and potential implant failure. *Adapted from* (Huang et al., 2024) *with permissions*. Abbreviations: TCF: T-cell factor, LEF: lymphoid enhancer factor, LPS: Lipopolysaccharide, TLR4: Toll-like receptor 4, TLR-2: Toll like receptor 2, RANK: Receptor Activator of Nuclear Factor kappa-B, ROS: Reactive oxygen species.

If M1 macrophage polarization is mediated, inflammatory process will be initiated to clear pathogens (Ma et al., 2014; Zhu et al., 2021). They will secrete high levels of pro-inflammatory cytokines such as TNF- α, IL-1β, IL-6 and inducible nitric oxide synthase (iNOS) (Zhang Y. et al., 2025; Zhou et al., 2025; Ma et al., 2014; Wang et al., 2019a; Quan et al., 2020). Such cytokine release is greatly associated with osteoclast activation *via* the RANK/RANKL/OPG signaling pathway thus leading to osteolysis and bone resorption. Pre-osteoclast differentiation to osteoclasts is mainly mediated by the IL-1β release from the M1 macrophages (Kheder et al., 2023). The prolonged M1 response results in chronic inflammation that is highly correlated with fibrous capsule formation, osteolytic loosening, tissue injury and eventually, implant failure as in peri-implantitis (Zhou et al., 2025; Moon et al., 2024; Ma et al., 2014; Zhu et al., 2021; Zhang et al., 2019). Conversely, if M2 macrophages are activated, anti-inflammatory cytokines will be released such as IL-10, IL-4, TGF- β, Arginase-1 (Arg-1) and vascular endothelial growth factor (VEGF) (Zhu et al., 2021; Quan et al., 2020). These factors facilitate the migration, localization and osteogenic differentiation of mesenchymal stem cells (MSCs) to osteoblasts, thus supporting new bone formation. For that, a rapid and timely switch to M2 phenotype is needed for successful ossiointegration (Zhou et al., 2023; Zhou et al., 2025; Ma et al., 2014; Wang et al., 2019a), as M2 polarization correlates with higher bone to implant contact. At the molecular level, the macrophage switch and the competition between bone formation and resorption is driven by specific intracellular pathways. As M2 macrophage can promote osteogenesis through the Wnt/β-catenin pathway, chronic inflammation triggers N kβ pathways. Figure 5 shows how inflammatory stimuli such as lipopolysaccharides (LPS) and pro-inflammatory cytokines activate TLR and RANK signaling ultimately leading to osteoclasenogenis and bone loss. For more comprehensive analysis about the complex immune dysregulation involved in this process, Li et al. (2024) present a deeper explanation of macrophage polarization patterns in the pathogenesis of peri-implantitis (Li et al., 2024). Moreover, for more detailed review about the signaling mechanisms refer to Huang et al. review paper (Huang et al., 2024).

Comparatively, strategies that bias macrophages toward M2 polarization show promise for osseointegration, yet they risk suppressing necessary early inflammatory responses. The unresolved debate is whether immune-instructive surfaces can achieve a stable balance without tipping toward chronic inflammation or impaired pathogen clearance. Macrophage polarization is highly dependent on the implant’s surface characteristics such as topography, wettability and surface chemistry. The following section explains how surface characteristics can induce macrophage switch as well as other immune cells.

Beyond chronic inflammation, common clinical complications like pain and allergic reactions have been reported (Siddiqi et al., 2011). These reactions as often due to type IV delayed type hypersensitivity reactions. This occurs when metals ions or Ti ions from a damaged Ti passivation layer is released to the near implant environment and acts as a hapten that binds to endogenous proteins. Such complexes are recognized by antigen presenting cells (APCs), which then activate T cells triggering a localized immune response characterized by pain, swelling and tissue redness. This hypersensitivity reaction and side effects can be overcome by different surface modification approaches (Goutam et al., 2014; Lozano et al., 2022) Figure 6 illustrates the Ti hypersensitivity reaction with explaining how surface modification can improve bone formation.

**FIGURE 6:**
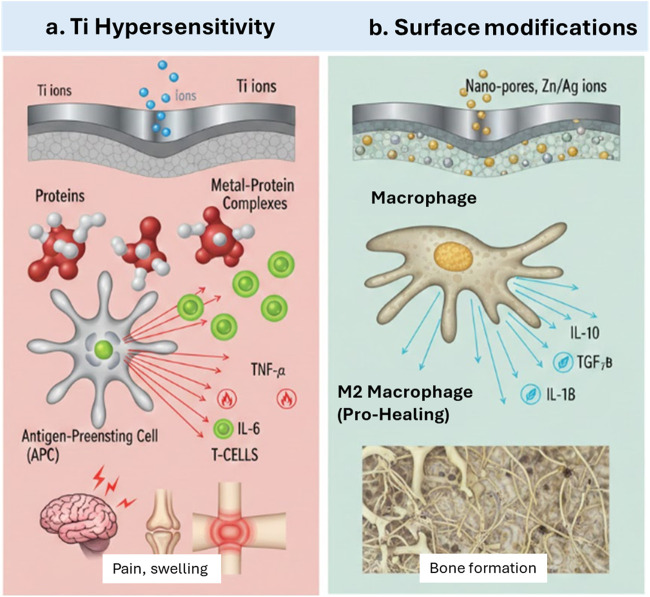
Represents the immune response to Ti implants in two step processes, influenced by the surface properties and host reactions. **(a)** represents the type IV delayed type hypersensitivity mechanism, in which corrosion causes leaching of Ti ions, which acts as haptens by binding to proteins. Thereby creating antigenic complexes that are taken up by APCs. It will be represented to T cells, thus inducing an immune cascade and proinflammatory cytokine release, thus leading to clinical pain and swelling. **(b)** illustrates the role of surface modification on assisting bone formation.

### 2.4 Impact of surface modifications on inflammatory response beyond macrophages

While macrophages have the main role in implant success or rejection, they are still a part of a broader immune population whose recruitment and activation are critically modulated by the implant interface. For example, in the initial injury response immune cells like lymphocytes are infiltrated. Studies using whole transcriptome analysis confirmed the expression of lymphocytes proliferation and cell mediated immunity signaling pathways in peri implant tissues more specifically in failed implants. In such cases, it’s been reported the increased infiltration of total lymphocytic cells. Surface modification that manages inflammation can indirectly modulate the activity and concentration of these lymphocytes (Kheder et al., 2023). The macrophage phenotype is known to regulate helper cell population *in vivo*. For that, by achieving M2 dominance, the functionalized implant surface actively minimizes the pro-inflammatory cytokines this preventing the recruitment and activation of T helper cells thus, promoting the necessary environment for long term implant success (Kheder et al., 2023; Dhaliwal et al., 2019).

Neutrophils are the first responders along with mast cells and dendritic cells, their initial response and activation is triggered by the presence of Ti particles. For that, modified Ti surfaces can significantly decrease the pro-inflammatory neutrophil behavior resulting in reduced cytokine production. Other cells like dendritic, mast and granulocytes are present in peri-implant tissue, contributing to the initial inflammation (Kheder et al., 2023). Surface modifications can interfere with the molecular machinery that cause inflammation, mainly through regulating the expression of some key signaling molecules. For example, functional antioxidant coatings with nanoparticles incorporation. Nanoparticles in this case act as nano-enzymes with superoxide dismutase like activity that act to prevent any inflammatory effect. Such modifications are needed, especially in cases where ROS are excessive, mainly due to systemic conditions such as diabetes. Other components like bio-inspired polymers such as polydopamine act as a radical scavenger limiting the generation of ROS and suppressing inflammation (Abaricia et al., 2020; Kheder et al., 2023).

Here, antioxidant and bio-inspired coatings mitigate ROS and inflammation, but questions remain about their durability under mechanical stress and systemic conditions such as diabetes. While these coatings reduce acute inflammation, their long-term effect on adaptive immunity and implant survival is still controversial.

Additional approach includes the NF-B signaling pathway inhibition, being one of the major molecular regulators of the inflammatory response. This can be achieved by different modification methods, including the chemical pretreatments for example, when salinization is applied to Ti surface before functionalization, it can significantly reduce the signaling pathways unlike pristine Ti surface that causes acute inflammation. Further approach is to incorporate therapeutic molecules like quercetin which terminates infection induced excessive inflammation mainly by regulating macrophage polarization by the signaling pathway (Zhang et al., 2019; Wang et al., 2019b; Lee et al., 2016; Zhou et al., 2025). Moreover, surface modification can actively suppress the chemoattractant molecules responsible for recruiting inflammatory cells. For example, surface modifications that has hydrophilic Ti surfaces or nanostructured surfaces downregulate the pro-inflammatory genes such as TNF-alpha, IL-1B and IL-6. and reduce the gene expression of Ccl2, Ccl3, Ccl4, Ccl5 and Ccl17 which are mediators of leukocyte recruitment (Abaricia et al., 2020; Kheder et al., 2023; Zhang et al., 2019; Wang et al., 2019b; Lee et al., 2016; Zhou et al., 2025; Yang et al., 2025; Zhang et al., 2018b).

There are some indirect surface functionalization ways to promote bone regeneration, such as using angiogenic peptides like K15 sequence in DOPA-P1@P2 which directly promotes angiogenesis by increasing the VEGF and FGF-2 expression and enhancing tube formation in HUVECs (Wang et al., 2019a). Nanotubular TiO has been shown to increase macrophage secretion of VEGF to accelerate endothelization (Wang et al., 2018).

Despite these promising findings, most evidence remains limited to *in vitro* or small animal models. Translation to clinical practice is constrained by short study durations, simplified biofilm models, and lack of patient heterogeneity. These limitations highlight the need for standardized protocols and long-term clinical validation.

## 3 Conventional strategies for dual functionality

### 3.1 Topographical modifications

Modifying the implant surface at the macro, micro and nanoscales is a key strategy. This section addresses the role of topographical modifications such as using nanostructures like titanium dioxide (TiO_2_) nanotubes, nanopores and roughness aiming to enhance osseointegration while preventing bacterial contamination.

Among nanoscale modifications, TiO_2_ nanotubes fabricated using the electrochemical anodization (EA) have gained importance (Wang et al., 2019a; Wang et al., 2018; Li et al., 2018). EA involves immersing the Ti implant (as the anode) and a non-target metal into an appropriate electrolyte (water with fluoride ions) with the application of suitable voltage. This method allows for the predictable fabrication of self-ordered TiO_2_ nanotubes on the surface thus creating a desirable dual micro/nano topography (Li et al., 2018; Narendrakumar et al., 2015; Guo et al., 2021). The resulting nanotubular architecture provides dual benefits as reported in multiple studies, as the structure of nanotubular TiO_2_ mimics the porous structure of the natural bone tissue, which is favorable to osteogenic differentiation. Studies demonstrated that the diameter or dimension of the nanotube significantly dictates cell functionality and fate (Su et al., 2018; Ding et al., 2015; Sabino et al., 2021; Roguska et al., 2018). For example, 15 nm is cited as the optimal length scale of surface topography for cell adhesion and differentiation. *In-vivo* studies have shown that TiO_2_ nanotubes enhance bone biding with reports indicating a nine-fold improvement in bone bonding in rabbits compared to non-treated Ti. The rate of mineralization associated with TiO_2_ nanotube surface is approximately three times the non-treated Ti surface (Wang et al., 2023; Narendrakumar et al., 2015; Choi et al., 2015).

Moreover, TiO_2_ nanotube exhibits antibacterial capacity and drug delivery capability. As reported TiO_2_ nanotubes can reduce the initial colonization and adhesion of *S. epidermidis*. However, the antibacterial effectiveness of bare TiO_2_ nanotextured surface alone, without additional functionalization, yields mixed results in the literature (Li et al., 2018; Narendrakumar et al., 2015; Martinez-Marquez et al., 2020; Guo et al., 2021). Though, some studies show a positive relationship for example, TiO_2_ nanotubes alone were reported as not being antimicrobial. However, the structure of the nanotubes allows them to act as a drug nano reservoir for the local delivery of therapeutics, including antibiotics, proteins and anti-inflammatory drugs. Which makes the TiO_2_ nanotubes topography versatile platform for achieving the dual functionality needed, mainly by incorporating additional agents (metal ions, bioactive molecules, antimicrobial agents…) (Li et al., 2018; Narendrakumar et al., 2015; Martinez-Marquez et al., 2020).

Using Ti surface with nanopores was reported in the literature as well, such porous formation is achievable through the EA method discussed previously. These porous formations were reported to have a crucial role in enhancing bioactivity and structural stability (Narendrakumar et al., 2015; Guo et al., 2023). As anodized nanostructures enhance corrosion resistance that is mainly attributed to the thickened protective barrier. It was reported in one study that EA facilitated the growth of nanostructures while preserving the underlying micro-scale features which resulted in a desired dual micro-nano topography. This type of combined topography is important as micro-roughness is the gold standard for early osseointegration and stability, while the nanostructures enhance bioactivity. Nanoporous Ti surfaces have demonstrated a positive effect of osteoblasts, inhibiting osteoclastogenic action and promoting osteogenic cytokines. The dual functionality using Ti nanoporous surfaces is achieved as this hollow nature of the pores act as an ideal nanoscale reservoir for local drug loading and release, this is the primary mechanism for achieving the dual function (Guo et al., 2023; Mukaddam et al., 2023; Guo et al., 2021; Pierfelice et al., 2022). As in nanotubes, nanopores can be loaded with therapeutic agents such as antibiotics, or antibacterial ions/nanoparticles like silver or gallium. Nanoparticles incorporation has been shown to have extended antibacterial capacity against *S.aureus*. Gentamicin loaded nanostructures, inhibited both *S. aures* and *S. epidermis* proliferation while promoting mesenchymal stromal cell activity (Gulati et al., 2020; Guo et al., 2023). Target bioactivity can be achieved as well by loading specific growth factors and proteins such as fibroblast growth factor 2, this has been shown to significantly upregulate the proliferation, adhesion and extracellular matrix formation of human gingival fibroblasts thus enhancing soft tissue interaction. Furthermore, nano-engineered surfaces such as anisotropic nanopores have demonstrated the ability to selectively modulate cell response without relying on drug loading. These surfaces immunomodulate macrophage growth while maintaining osteoblasts and fibroblasts. This selective modulation supports timely tissue healing and prevents bacterial access through establishing a firm soft tissue barrier (Gulati et al., 2020; Guo et al., 2023; Ogura et al., 2022).

There are other nanostructure modification methods such as nitriding and nanoparticle deposition, nitriding adds a nanocrystal titanium nitride layer which improves the hardness and chemical resistance thus providing corrosion control. Plasma spraying and alkali heat treatment are further methods that deposit nanoparticles and nanoparticles to form a nanostructured layer that provides corrosion protection. This type of modification provides a nanoparticle layer that enhances the resistance against tribocorrosion and chemical stability. Further approaches like simple chemical and heat treatment that can be used to create nanostructured sodium hydrogen titanate later which can allow the incorporation of bioactive ions leading to dual functionality (Pierfelice et al., 2022; He et al., 2022; Guo et al., 2023).

Surface roughness is a key parameter that determines the interaction of the implant with bone cells and bacteria (Pan et al., 2021; He et al., 2022; Guo et al., 2023). Historically, surface modification efforts focused on introducing microroughness to enhance cellular contact and osseointegration (Almas et al., 2019; Rupp et al., 2018). It’s been reported that moderately rough Ti surfaces (between 1 and 2 µm) have been shown to have a superior osseointegration (Rupp et al., 2018; Rosa et al., 2012). The positive effect of surface roughness on bone integration is widely acknowledged. Microrough topographies provide osteoblasts with the structural indications needed to promote tissue regeneration and new bone formation. Common techniques used to create microroughness include grit-blasting, acid etching or combination of both (Bevilacqua et al., 2018; Boyan et al., 2017). For example, micro-rough surfaces can be generated by sand blasting followed by acid etching or by dual acid etching. Although micro-roughness enhances osseointegration, it presents a challenge concerning bacterial control. Increased bacterial retention is correlated with higher degree of roughness. Rough surfaces are difficult to clean and can facilitate rapid regrowth of biofilm. Roughness at the microscale level is generally understood to increase the potential for bacterial adhesion. To resolve the need for enhanced osseointegration (favors micro-roughness) and reduced bacterial adhesion (disadvantaged by micro-roughness). For that, research shifted toward incorporating nanoscale features. Current research highlights the importance of combined beneficial effects of nano-scaled topography and the implant surface wettability at the bone interface. By incorporating roughness at three different scales: macro, micro and nano, the implant can exhibit similar properties to the bone. The combination of micron and submicron scale structure is required for synergistic osteoblast responses to substrate surface energy and topography (Rosa et al., 2012; Rupp et al., 2018).

Nano-topography play a significant role in bacterial adhesion, for example, mechanical force cause by high aspect ratio nanostructured surface like in nanopillar, spikes or nanospheres can cause physical rupture in bacterial cells, leading to bacterial death and reducing biofilm formation, this mechanism is known as contact killing. Nanospikes can be fabricated using alkaline hydrothermal treatment, thus can be used to kill bacteria thus achieved a dual benefit. Studies have indicated that bacterial adhesion is gradually prevented on the nanoscale (10–100 nm) but increases with roughness at the microscale (Matsubara et al., 2020; Costa‐Berenguer et al., 2018; Safaei et al., 2024).

### 3.2 Inorganic modifications and ion doping

A further approach to achieving dual functionality is by incorporating inorganic trace elements and metallic ions directly into the surface coating or the bulk material. Since a single elemental modification often fails to provide all the required properties of optimal implant performance, co deposition has emerged as a major area of research (Hameed et al., 2022; Zhang et al., 2025a; Salaie et al., 2023).

Silver is one of the most widely studied inorganic antimicrobial agents, valued for its antibacterial properties and its use in different dental materials including acrylic resins, composite resins and materials for guided tissue regeneration. Surface modification with silver often involves the incorporation of dissolved silver fractions. This dissolved silver has been shown to significantly decrease bacterial growth in suspension (Qin et al., 2015; Yang et al., 2023). For example, the dissolved silver released into brain heart infusion broth from silver coated discs was reported to inhibit the growth of *streptococcus mutans* (Salaie et al., 2023). Furthermore, nanocrystalline silver coating applied to Ti surface has demonstrated a strong and localized antibacterial effect (Gosau et al., 2016). Silver ion coating was reported to have antibacterial activity against common peri-implantitis pathogens such as *Porphyromonas gingivalis, Prevotella intermedia,* and *Aggregatibacter actinomycetemcomitans*. Silver is frequently investigated for its dual action potential, combining antibacterial activity with osteogenic compatibility (Chandran et al., 2025). A highly promising dual strategy involves co-doping with other metals. For example, synergistic effects in enhancing both osteogenic activity and antibacterial ability have been observed through the dual implantation of Zn/Ag ions which form micro-galvanic couples on Ti (Okuzu et al., 2017; Yu Y. et al., 2017; Zhang et al., 2025a). Another study developed a multifunction inorganic composite coating on Ti implants, by doping porous Ti with silicon and silver, such modification lead to achieving the dual functionality needed for any implantable material (Zhao et al., 2022).

Copper is an essential element utilized in coatings due to its recognized antimicrobial ability and potential benefits for biological processes. It can be integrated into Ti through alloying (TiCu implants) or doping into surface coatings such as magnesium phosphate (MgP) supraparticles (Liu et al., 2022; Lanzino et al., 2025). Copper functionalized Ti significantly reduces the survival of surface adhered *P. gingivalis* bacteria (Astasov-Frauenhoffer et al., 2019). Ti-Cu alloys provide an effective and sustained bactericidal effect against oral bacterial like *S. mutans* and *p. gingivalis* and inhibit bacterial adhesion and biofilm formation. The mechanism relies on the release of Cu ions; this release acts as a safe zone that protects the surrounding environment against bacteria which is needed for implant healing (Hameed et al., 2022). Coatings that have incorporated copper such as MgPCu have shown strong antibacterial efficacy, reducing the number of CFU of *E. coli* by over 99% and *S. aureus* by over 97%. Ti-Cu alloys have demonstrated high biosafety and biocompatibility while exhibiting antimicrobial effects (attributed to the Cu^2+^ ions release) (Lanzino et al., 2025). The cytotoxicity of copper relies significantly on its concentration. Low molar concentration can be beneficial for dual functionality. It has been reported that copper combined with hydroxyapatite showed superior cell viability compared to bare Ti and Ti/Cu samples. Additionally, copper can be combined with zinc in coatings such as Cu/Zn co-implanted Ti nitride coatings, which showed an enhanced antibacterial ability to approximately 99.12% against *E. coli* (Li et al., 2019).

Zinc is an essential trace element involved in many biological processes, including cell development, DNA synthesis and enzyme activity (Jin et al., 2014). Its incorporation usually aids in the dual functionality of Ti implants. Zn is widely believed to stimulate bone formation, studies involving Zn implanted Ti surfaces have shown that Zn doping significantly promotes the proliferation, initial adhesion, spreading activity, alkaline phosphatase activity, collagen secretion and ECM mineralization of rat mesenchymal stem cells. Moreover, incorporating Zn into Ti implant surface has been reported to enhance new bone formation (NBF) and bone to implant contact (BIC) (Jin et al., 2014; Kellesarian et al., 2017). For example, one study observed a significantly higher BIC and shear strength for Zn-coated Ti surfaces compared to un-coated surfaces. Although the incorporation of Zn significantly improves the NBF, still a lot of standardization and long-term clinical studies are needed to confirm the long-term success of Zn incorporated Ti dental implants (Kellesarian et al., 2017). Zinc doped materials exhibit antibacterial activity through the gradual and continuous release of Zn^2+^ ions, it’s been reported to have a strong antibacterial effect against both *E. coli* and *S. aureus*. To achieve the best performance, zinc is frequently utilized in multifunctional ion pairings, for example, Zn/Ag ions was reported to improve both osteogenic activity and antibacterial ability. Furthermore, Zn/Mg co-implantation showed strong bacterial inhibition (Han et al., 2024).

Strontium (Sr) is primarily incorporated into titanium surfaces to enhance bone bonding and accelerate osseointegration. Sr-modified coatings, such as Ti-Sr-O applied using industrial scale magnetron sputtering, display a surface morphology characterized by a porous cauliflower-like nanostructure. Sr doping using simple chemical treatment (NaOCl-Sr washing) enhances osseointegration (Okuzu et al., 2017). Ti-Sr-O coated implants demonstrated a higher mean BIC % and higher NBF % in defined regions of interest compared to uncoated Ti surfaces. Furthermore, Sr-doped implants showed significantly higher fluorescence density values compared to control groups at 2- and 4-weeks post-implantation, which indicates increased active bone metabolism and bone proliferation (Urangoo et al., 2022). Sr positive effect on osseointegration is gained through the release or Sr ions from bioactive Ti metal which promotes early bone bonding. Strontium is typically combined with other elements to achieve dual functionality, for example, Sr/Zn/Ca coating has been shown to improve osseointegration by regulating macrophage polarization, indicating a complex biological interaction that supports healing (Zhang et al., 2025a; Okuzu et al., 2017).

### 3.3 Organic/biological functionalization

One of the conventional approaches that are commonly used is based on the utilization of biomolecules to simultaneously enhance osseointegration and provide antibacterial protection. This strategy involves modifying the material surface using peptides (including antimicrobial peptides) and various growth factors (Sharan et al., 2018).

Peptides are biomolecules that are frequently used in surface modification due to their specificity, allowing the engineering of surfaces that promote specific biological and antimicrobial responses (Gómez et al., 2023). To achieve the desired balance between enhancing osseointegration and preventing bacterial contamination, a key strategy involves engineering peptides that combine multiple functions. One of these is chimeric peptides These structures typically fuse an antimicrobial sequence with a Ti- binding sequence (Liu et al., 2016; Drexelius et al., 2023). Novel chimeric peptides were designed, for instance, by fusing the antimicrobial peptide MGD2 with titanium-binding sequences (TiBP1, TiBP2, or TBP-1) (Liu et al., 2016). The objective is to inhibit initial colonizer adhesion and subsequent biofilm formation. Studies confirm that using a chimeric peptide (JH8194, an antimicrobial motif, coupled with minTBP-1, a binding motif) connected by rigid linkers can significantly improve both the binding affinity and the antimicrobial abilities on Ti surfaces (Liu et al., 2016). These chimeric peptides demonstrate promising antibacterial activity, even against drug-resistant strains, suggesting their utility as anti-biofilm compounds. Coating titanium plates with these chimeric peptides (Chim1-Chim3) significantly reduced the number of viable bacteria colonies, such as *Bacillus subtilis*, compared to unmodified plates (Chen et al., 2021). Fusion Peptides are another type that have been developed since they exhibit antimicrobial activity, biocompatibility, angiogenic activity, and osteogenic activity without the need for external stimuli. This strategy proved effective against a panel of clinically important bacteria, including *S. aureus*, *E. coli, Porphyromonas aeruginosa*, and *MRSA* (Chen et al., 2021).

Antimicrobial peptides (AMPs) offer a promising approach for surface modification, often showing low toxicity toward multicellular organisms (Subh et al., 2020). One of these is the cathelicidin peptide when functionalized on titanium surfaces has been shown to yield a platform that is biocompatible with osteoblasts while maintaining its anti-bacterial activity (Gómez et al., 2023). Other type of peptides include the Caerin peptides which is derived from amphibian skin, it has been reported to exhibit antimicrobial effect. It was reported that Coating titanium plates with the caerin 1.9 peptide (F3 group) effectively inhibited bacterial growth in *in-vivo* rabbit mandible. Furthermore, the F3-coated titanium plate was reported not to show any signs of triggering the immune repose of causing inflammation, thus suggesting favorable biocompatibility (Long et al., 2024).

Peptide-drug conjugate is another modification strategy that involves covalently conjugating peptides with other therapeutic agents to achieve synergistic dual functionality. One study reported the fabrication of a novel antimicrobial and osteogenic conjugate, osteogenic growth peptide (OGP) - ciprofloxacin (CIP) was synthesized by coupling the OGP with the antibiotic CIP. When immobilized on Ti substrates, this compound provided significant antibacterial rates, reaching 93.6% against *S. aureus* and 95.1% against *E. coli* after 6 h (Liu et al., 2018). Additionally, the Ti-OGP-CIP substrate enhanced osteoblast differentiation, exhibiting increased Alkaline Phosphatase (ALP) activity and mineralization compared to controls. The OGP-CIP modified substrate also demonstrated sustained release for over 7 days, with slightly improved release kinetics in acidic environments (which can occur during bacterial infection or osteoclastogenesis) (Liu et al., 2018).

Peptides that promote cell adhesion are frequently used to stimulate host tissue interaction and accelerate osseointegration (Trino et al., 2018). One of the widely used peptides is the Arg-Gly-Asp (RGD) peptide which can be covalently immobilized into Ti surfaces. This modification stimulates osteoblast-like cell adhesion, proliferation, and differentiation. Studies comparing linear (l-RGD) and cyclic (c-RGD) RGD-modified surfaces showed that RGD peptides promote increased cell coverage and higher alkaline phosphatase expression, suggesting a positive bone response. Other studies reported the modification with biomolecules like Type I Human Collagen (T1HC) as it showed improved cell adhesion, spreading, and growth (Liu et al., 2018). Other biomolecules used include dentin matrix protein 1 (DMP1) peptides, which have been shown to enhance osteogenic differentiation and form minerals with a Ca/p ratio near that of hydroxyapatite. Furthermore, chimeric proteins, such as one combining fibronectin fragments (FN9-10) with an elastin-like polypeptide (FN9-10ELP), have been developed to enhance human mesenchymal stem cell proliferation, adhesion, and osteogenic differentiation markers (Col I, RUNX2, OPN, OCN, BSP, and TAZ) over extended periods (Heller et al., 2018).

Growth factors are critical for triggering osteo-induction and supporting bone regeneration. They are typically incorporated onto implant surfaces to provide localized delivery. BMPs, particularly BMP-2 and BMP-7 are well known Osteo-inductive agents that promote bone formation by stimulating the differentiation of MSCs (Lee et al., 2012). BMP-2 can be chemically coupled to the Ti surface using linking agents. Immobilization of recombinant human BMP-2 (rhBMP-2) on Ti implants has been shown to induce integrating amounts of peri-implant trabecular and cortical bone. A common strategy for dual functionality involves combining BMPs with antimicrobial agents in a spatially or temporally controlled manner. A strategy was developed where BMP-2 was immobilized exclusively on the implant body to promote bone regeneration, while the antimicrobial agent chlorhexidine (CHX) was immobilized at the implant collar using a thermosensitive hydrogel delivery system (Chuang et al., 2025). This configuration aims to provide localized antibacterial protection where needed, while simultaneously enhancing osseointegration in the bony defect. Another approach developed a dual drug-eluting system by sequentially immobilizing gentamicin sulfate (GS) and BMP-2 onto heparinized titanium implants. This system served as an excellent platform to develop next-generation implants by simultaneously delivering antibiotics and osteo-inductive proteins (Lee et al., 2012; Chuang et al., 2025; Kämmerer et al., 2020).

**FIGURE 7:**
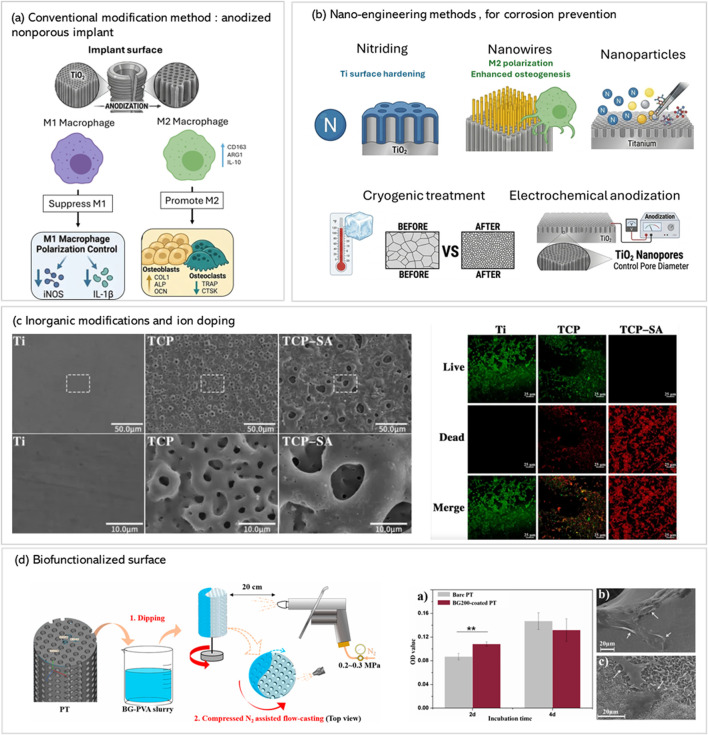
Multifunctional surface modification strategies for titanium implants **(a)** Anodized TiO_2_ nanopores modulate macrophage polarization, promoting M2 phenotype and osteogenic signaling (COL1, ALP, OCN), while suppressing osteoclast markers (TRAP, CTSK). **(b)** Nano-engineering techniques such as nitriding, nanowire deposition, and cryogenic treatment enhance corrosion resistance and mechanical stability under physiological stress. **(c)** Inorganic modifications and ion doping (e.g., Ag +Si incorporation) enhance antibacterial efficacy, as shown by SEM images comparing untreated (Ti), to surface modified samples with more bacterial inhibition in the modified samples. **(d)** Biofunctionalization *via* flow-casting of BG-PVA slurry onto porous titanium (PT) scaffolds yields uniform coatings that preserve macroporosity, improve cell viability (as shown in OD assays at 2 and 4 days), and support favorable cell morphology and adhesion, indicating enhanced biocompatibility and potential for clinical translation. *Adapted from* (Astasov-Frauenhoffer et al., 2019; Park et al., 2021; Gao et al., 2023; Moon et al., 2024; Zhao et al., 2022) *with permissions*.

To ensure long-term implant success, promoting blood vessel formation (angiogenesis) is often necessary alongside osteogenesis, especially in porous scaffolds. For that Vascular Endothelial Growth Factor (VEGF) is a highly studied angiogenic factor. High Mobility Group Box 1 (HMGB1) is another factor that shows chemotactic potential for endothelial cells, sometimes exceeding that of VEGF *in vitro*. HMGB1, often combined with CXCL12 (chemokine ligand 12), can be incorporated into polymers such as Polycaprolactone (PCL) coatings on porous titanium implants to support angiogenesis in bony defects. The use of polymers like PCL allows for direct release of these factors over time, thereby enhancing concentration in the area of interest. Growth factors can also be combined within a delivery vehicle, such as incorporating BMP-2 and VEGF into fibrin glue used to functionalize 3D-printed porous titanium scaffolds, thereby stimulating both angiogenesis and osteogenesis (Li et al., 2020; Matena et al., 2015). Figure 7 summarizes the different conventional modification methods.

### 3.4 Advanced macroporous structures and 3D-printing

While nano-scale modifications are centered on cellular signaling, macro-scale structural modifications through additive manufacturing have transformed the mechanical integration of implants. Recent advances in 3D-printed porous Titanium (Ti) implants provide a promising alternative to conventional threaded implants. The porous structure enables a modifiable elastic modulus that precisely replicates the properties of human bone, thus overcoming the ‘stress shielding’ phenomenon and associated marginal bone loss (Chakraborty et al., 2023). In addition, the porous structure enables a dramatically increased surface area that promotes deep bone ingrowth (osseointegration) and supports osteoblast adhesion and proliferation (Iezzi et al., 2024). Using Selective Laser Melting (SLM) or Electron Beam Melting (EBM), these advanced systems enable a biomimetic scaffold that supports excellent primary stability and long-term biological fixation compared to conventional solid Ti implants.

## 4 Advanced systems: smart and responsive Ti

The advancement of smart and responsive Ti surfaces in implant technology is needed to achieve the needed balance between effective antibacterial activity and enhanced osseointegration. Such advanced systems use external stimuli response (ESR) or smart stimuli responsive strategies (SSR), mainly incorporating nanoscale components and bioinspired design to modulate properties on demand or in response to physiological microenvironment.

### 4.1 SSR coatings: surfaces triggered by infection signals

SSR coatings are designed to use therapeutic effects or promote regeneration in response to specific alterations in the pathological microenvironment, such as bacterial infection or chronic inflammation. Such changes act as a trigger to release drug or to activate surface function (Zhang et al., 2024; Hu et al., 2023; Wei et al., 2019).

#### 4.1.1 Response to low pH

Bacterial infection sites are known for their mild acidic microenvironment with pH range from 5 to 5.5. This pH is different from the normal physiological pH which is around 7.4, this is mainly due to the metabolic production of substances such as lactic acid and acetic acid (Zhang et al., 2024; Hu et al., 2023; Wei et al., 2019).

Many strategies use this pH drop to trigger the release of antibacterial agents, mainly achieved by incorporating pH sensitive material and coatings. One of these methods is using materials like synthetic polymers as in poly methacrylic acid (PMAA) can be used for a Pandora’s box system where the polymer swells to seal drug loaded Ti nanotubes in physiological condition. But collapse in acidic environments and release the drugs/agents like antimicrobial peptides (Wei et al., 2022; Ahmed et al., 2019). This design verified the pH-dependent release and anti-bacterial effect *in vitro* and *in vivo*, alongside satisfying pro-osteogenic activity. Additionally, the protein layer of silk fibroin coatings loaded with silver nanoparticles and gentamicin has been reported to exhibit smart release profiles in response to pH variations. The responsive mechanism is associated with molecular conformation changes of silk fibroin in response to decreased pH, weakening the binding force of drugs and coating compactness. Further approach that involves acid sensitive chemical bonds, such as metal ion coordination bonds, break down in acidic conditions. For instance, coordination polymers blocking drug release from Ti nanotubes were successfully triggered to open and release antibiotics like vancomycin when the environment became acidic due to infection. Metal organic frameworks such as cobalt metal organic frameworks synthesis on Ti nanotubes, were reported to be able to decompose in the acidic environment thus releasing Co^2+^ ions which shift the environment to alkalinity. This action has been reported to induce both antimicrobial effects with promoting osteogenesis and MSCs osseointegration (Gao et al., 2023; Moon et al., 2024; Chakraborty et al., 2023; Iezzi et al., 2024).

#### 4.1.2 Responses to bacterial enzymes

Pathogenic bacteria secrete high levels of specific enzymes/virulence factors that can be targeted to achieve highly specific drug release (Hu et al., 2023). One of these is hyaluronidase which is secreted by bacteria like *S.aureus* and degrades hyaluronic acid (HA) (Wei et al., 2019; Ahmed et al., 2019). For that, coatings containing HA-antibiotic conjugates like HA-gentamicin and chitosan multilayers decompose upon exposure to hyaluronidase, releasing the antibiotic and deferoxamine was reported. This approach yielded antibacterial effects and pro-regenerative properties. Further study used a coating utilizing a layer of sodium hyaluronate-catechol to cover ciprofloxacin CIP-loaded mesoporous polydopamine PDA achieved bacteriostatic efficacy through the synergy of hyaluronidase triggered CIP release and near infrared NIR light-triggered photothermal effect. MMP-9 is another marker that is secreted by osteoclasts and immune cells during tissue remodeling and inflammation. A dynamic coating was developed by co-immobilizing the antimicrobial peptide GL13K and an MMP-9 cleavable peptide onto titanium. This multi-peptide coating showed potent anti-biofilm activity and demonstrated MMP-9 dependent release of GL13K, offering a means of synchronization between biomaterial cues and tissue responses during bone remodeling. Moreover, exogenous product of *S.aures* was used as a trigger to degrade poly-L-glutamic acid films on silver containing mesoporous silica nanoparticles, resulting in the release of silver ions to eradicate infection and promote osseointegration (Intravaia et al., 2023; Wei et al., 2022).

### 4.2 Synergistic and photo-active systems

Photo-stimulation, particularly using NIR light, is widely employed due to its non-invasiveness and deep tissue penetration. These strategies influence photothermal therapy (PTT) and photodynamic therapy (PDT) synergistically, often combined with chemical release mechanisms, to balance antibacterial activity and osteogenesis. PTT converts light into localized heat using photothermal agents, such as (PDA), red phosphorus (RP), or carbon-based materials. Temperatures above 45 °C can eliminate bacteria by rupturing cell membranes and denaturing proteins. Temperatures ranging from 40 to 42 °C can also promote osteogenic differentiation. PDT on the other hand utilizes photosensitizers (Ps) to generate ROS such as superoxide and hydroxyl radicals, which chemically destroy bacteria and biofilms. TiO_2_ is inherently photo-sensitive but requires modification like chemical doping or incorporating photosensitive molecules, to be activated by safer visible or NIR light instead of harmful UV light (Intravaia et al., 2023; Zhao et al., 2023; Liu et al., 2024; Yang et al., 2024).

**FIGURE 8:**
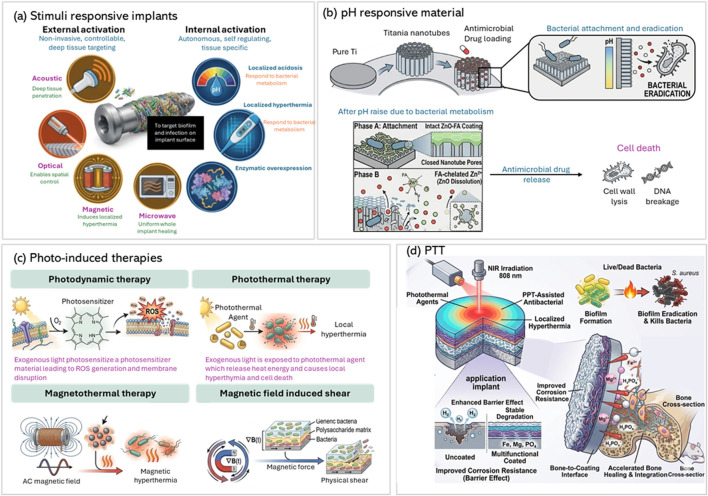
Advanced stimuli-responsive strategies for implant surface modification to combat infection and enhance regeneration. **(a)** Classification of environment-responsive therapeutic platforms into exogenous (photo, magnetism, microwave, ultrasound) and endogenous (acidic pH, temperature, enzymatic activity) stimuli, enabling targeted antibacterial activation at infected implant sites. **(b)** Schematic of a pH-responsive titanium nanotube system loaded with vancomycin and ZnO-FA coating, which dissolves under acidic conditions to release Zn^2+^ and antibiotics, achieving localized bacterial eradication. **(c)** Illustration of photo-induced therapies including photothermal therapy (PTT), photodynamic therapy (PDT), and magnetism-based approaches, which leverage exogenous stimuli to trigger reactive oxygen species (ROS) generation, hyperthermia, and biofilm disruption **(d)** Photothermal therapy schematic showing a multifunctional PEO/PDDF/GOH-coated implant that, upon irradiation, kills bacteria, prevents biofilm formation, and promotes osteogenesis while enhancing corrosion resistance and biocompatibility. *Adapted with modifications from* 8 **(a,d)** (Gómez et al., 2023), 8 **(b)** (Subh et al., 2020) and 8 **(c)** (Li et al., 2020).

Combining PTT and PDT is highly effective, as heat enhances the permeability of the bacterial cell membrane, improving the antibacterial effects of ROS and chemical agents. Triple Therapy including all PTT/PDT/Antibiotic was reported as well. In which coating using PDA as a binder for IR820 (photosensitizer) and Daptomycin achieved photothermal, photodynamic, and antibiotic triple therapeutic properties under NIR light, reaching over 97% antibacterial efficiency. Further studies reported the use of multifunctional coatings in which NIR light activates photothermal and photodynamic processes, generating ROS which in turn catalyze a secondary chemical reaction (Zhang et al., 2024; Wei et al., 2019). For example, TiO_2_ nanosheets doped with up-conversion elements (Yb/Er) coupled with L-arginine (LA) generate ROS that oxidize lactic acid to release nitric oxide The combined effects of heat, ROS, and nitric oxide eradicate biofilms, inhibit tumors, and promote angiogenesis and osteogenesis. Mesoporous PDA loaded with carbon monoxide (CO) nanogenerators (Fe_3_(CO)_12_) released CO gas upon NIR irradiation. The concept is summarized in Figure 8. The hyperthermia potentiated mild PTT, and the CO delivery alleviated infection-induced inflammation by shifting macrophage polarization towards the pro-regenerative M2 phenotype. Additional approach relies on incorporating red phosphorus (RP) film with IR780, this approach achieved synergistic PDT and PTT effects against biofilms. RP combined with calcium titanate formed a P-N heterojunction, enhancing photothermal and photocatalytic properties for high antibacterial efficacy while maintaining osteoconductivity (Gao et al., 2023; Hu et al., 2023; Ahmed et al., 2019).

### 4.3 Bio-inspired multifunctional coatings

Bio-inspired coatings integrate functions derived from nature to produce complex, multi-modal responses that achieve both antibiosis and regeneration (Intravaia et al., 2023). One of these approaches is mussel-Inspired PDA, inspired by mussel adhesion, PDA is widely used due to its strong adhesive properties, high photothermal conversion efficiency, and abundant active groups (OH group and NH_2_) that allow easy grafting of functional molecules. PDA can function as a photothermal agent itself while simultaneously acting as a grafting medium for antibiotics, photosensitizers, or cell-adhesive peptides (like RGD) to create multifunctional surfaces. Bio-functional peptides and chimeric peptides are other examples (reported in the previous section) however enzyme responsive peptides are other example of this smart modification, for example, a system combining GL13K AMP and MMP9-CP is a prime example of a bioinspired dynamic surface that responds to specific enzymatic signals related to bone remodeling processes, enhancing anti-biofilm activity while promoting osteoblast proliferation (Fischer et al., 2021). To ensure multifunctionality, coatings often integrate osteogenic components. Bioactive molecules, such as the cell adhesion sequence RGD, are often incorporated into photothermal or stimuli-responsive coatings to promote cell proliferation and osteogenic differentiation, addressing the challenge of potential cytotoxicity from antibacterial agents (Gao et al., 2023; Hu et al., 2023; Ahmed et al., 2019; Intravaia et al., 2023).

Current developments have moved towards the development of “smart” and highly efficient antibacterial coatings, which are designed to be less toxic and more antimicrobial (Akshaya et al., 2022). New developments include the application of iodine-supported titanium, which has shown considerable clinical success in inhibiting microbial growth without affecting thyroid function or biocompatibility (Akshaya et al., 2022). Additionally, zinc (Zn)-doped coatings prepared by plasma electrolytic oxidation (PEO) have proven to be a reliable method; these coatings not only improve mass loss by providing increased wear resistance but also decrease biofilm biomass by more than 60% by sustained ion release (Kheirmand-Parizi et al., 2024). Another new development in the field of coatings includes high-power impulse magnetron sputtering to prepare ultrathin silver (Ag) coatings. These 7 nm thick films exhibit strong bactericidal properties while maintaining safe and controllable levels of silver release that do not affect osteoblast cells, thus providing a good balance between the “race to the surface” of host tissue and microorganisms (Chahine et al., 2008).

### 4.4 Nanomaterial-based functionalization

Nanomaterials are integral to advanced titanium systems, leveraging their size-dependent properties to enhance both biological activity and responsiveness. Nano-phase materials can mimic natural tissue features, improving functions like wettability, topography, and surface energy. As discussed in the previous section, Ti nanotubes are highly recognized as promising platforms for localized and smart drug delivery. Their ordered tubular structure allows them to host drugs, antibiotics, or functional ions. Drug release kinetics from these nanotubes can be precisely controlled by modifying their morphology or by using responsive materials as nanogates at the orifice, such as pH-responsive polymers. Another approach that was reported is decreasing grain size to the nanophase level enhances the surface area, promoting improved protein synthesis, calcium mineral accumulation, and enhanced osteoblast proliferation and adhesion compared to conventional materials. TiO_2_ nanorod arrays have been shown to improve cell adhesion, proliferation, and osteogenic differentiation, thereby accelerating bone regeneration. Nanoparticles provide targeted functionality, for example, silver nanoparticles provide broad-spectrum antimicrobial activity, often used in conjunction with polymers or peptides for controlled release. They can be released in response to specific bacterial enzymes (Chen, 2022; Liang et al., 2024; Wang et al., 2016; Kumar et al., 2020). Table 1 represents a summary of different modification approaches.

**TABLE 1:** Summary of different modification approaches used.

Modification	Type	Mechanism	Antibacterial efficacy	Osseointegration impact	References
TiO_2_ nanotubes (EA anodization)	Topographical	Creates dual micro/nano topography; acts as drug reservoir	Mixed results bare; effective when loaded with antibiotics/ions	Increase Cell adhesion, differentiation; 9-fold bone bonding *in vivo*	Goutam et al. (2014); Lozano et al. (2022); Dhaliwal et al. (2019); Abaricia et al. (2020); Kheder et al. (2023); Zhang et al. (2019); Wang et al. (2019b); Lee et al. (2016); Zhou et al. (2025); Yang et al. (2025); Zhang et al. (2018a); Wang et al. (2019a); Wang et al. (2018); Li et al. (2018); Narendrakumar et al. (2015); Martinez-Marquez et al. (2020); Guo et al. (2021); Gulati and Ivanovski (2017); Somsanith et al. (2018); Zhang et al. (2021); Goutam et al. (2014); Lozano et al. (2022); Dhaliwal et al. (2019); Abaricia et al. (2020); Lee et al. (2016); Yang et al. (2025); Wang et al. (2018); Li et al. (2018); Martinez-Marquez et al. (2020); Gulati and Ivanovski (2017); Somsanith et al. (2018); Goutam et al. (2014); Lozano et al. (2022); Dhaliwal et al. (2019); Abaricia et al. (2020); Lee et al. (2016); Yang et al. (2025); Wang et al. (2018); Li et al. (2018); Martinez-Marquez et al. (2020); Gulati and Ivanovski (2017); Somsanith et al. (2018); Goutam et al. (2014); Lozano et al. (2022); Dhaliwal et al. (2019); Abaricia et al. (2020); Lee et al. (2016); Yang et al. (2025); Wang et al. (2018); Li et al. (2018); Martinez-Marquez et al. (2020); Gulati and Ivanovski (2017); Somsanith et al. (2018)
Nanoporous Ti (EA4 anodization)	Topographical	Dual micro-nano roughness; porous reservoir for drug/ion loading	Gentamicin/Ag/Ga nanoparticles inhibit *S. aureus*, *S. epidermidis*	Increase Osteoblast activity, cytokine release, fibroblast ECM formation	Su et al. (2018); Ding et al. (2015); Roguska et al. (2018); Narendrakumar et al. (2015); Choi et al. (2015); Kim et al. (2021), Su et al. (2018); Ding et al. (2015); Sabino et al. (2021); Choi et al. (2015); Kim et al. (2021), Ding et al. (2015); Choi et al. (2015); Kim et al. (2021); Su et al. (2018); Ding et al. (2015); Choi et al. (2015); Kim et al. (2021)
Surface roughness (grit blasting, plasma spray)	Topographical	Micro-roughness enhances early stability	Plasma-sprayed Ti reduces bacterial adhesion	Increase osteoblast proliferation, viability	Antonoglou et al. (2025), Petersen (2014)
Hydrophilicity/wettability tuning	Physicochemical	Alters surface energy, residual stress	Hydrophilic surfaces increase biofilm adhesion; compressive stress increase osteoblast proliferation without increasing the bacteria	Increase osteoblastic proliferation, bone contact	Hasani-Sadrabadi et al. (2021), Buga et al. (2021); Petersen (2014)
Surface charge (APTES modification)	Chemical	Positively charged hydrophobic layer	Eliminated infection in rat model; decrease bacterial adhesion	Histology: no inflammation, improved tissue response	Damiati et al. (2018), Parizi et al. (2022)
Photocatalytic Ti (UV activation)	Chemical/Physical	UV irradiation induces bactericidal ROS	decrease *P. aeruginosa*, *S. aureus* CFUs after 24 min UV	Maintains osteoblast viability	Safaei et al. (2024)
Antimicrobial peptides (GL13K hydrogel, chimeric TBP-1-RGDS-hBD3-3)	Biological	Peptide release disrupts bacterial membranes; recruits host cells	Inhibits streptococcal colonization, biofilm formation	Increase Osteoblast differentiation *in vitro*	Sousa et al. (2022), Diez-Escudero et al. (2022)
Antibiotic coatings (gentamicin nanoparticles, amphora pores)	Drug delivery	Sustained local release from coatings/nanopores	decrease *S. aureus* adhesion; prevented infection in rat model	Increase Cell adhesion, proliferation, bone integration	Gonzalez et al. (2020), Tardelli et al. (2023), Shen et al. (2020), Zhang et al. (2023)
Metal ion incorporation (Mn, Zn nanowires)	Chemical	Controlled ion release; osteogenic gene upregulation	decrease Gram-negative viability; Zn suppresses inflammation	Increase Osteogenesis, enhanced bone-to-implant contact	Soares (2024), Pereira et al. (2024)
Quaternary ammonium salts/oleic acid functionalization	Organic coating	Disrupts bacterial membranes	Strong inhibition of *E. coli* and *S. aureus* adhesion	Maintains biocompatibility	Pereira et al. (2024)
Immune-modulating coatings (antioxidant nanoparticles, polydopamine, quercetin)	Bio-inspired	ROS scavenging, NF-κB inhibition, macrophage polarization	Increase Pro-inflammatory cytokines, decrease leukocyte recruitment	Promotes M2 macrophage dominance, Increase bone formation	Zhu et al. (2021); Zhang et al. (2019); Quan et al. (2020); Zhou et al. (2023); Zhou et al. (2025)
Angiogenic peptide coatings (K15, DOPA-P1@P2)	Biofunctional	Increase VEGF, FGF-2 expression; angiogenesis	Indirect antibacterial *via* improved healing	Increase tube formation, vascularization, bone regeneration	Huang et al. (2024), Siddiqi et al. (2011)

## 5 The lab to clinic gap (translational challenges)

A significant difficulty in the translation from laboratory success to clinical reliability is the management of medically compromised patients. While surface modifications show promise in healthy *in vivo* models, systemic conditions such as uncontrolled diabetes, heavy smoking, and the use of antiresorptive medications (e.g., bisphosphonates) significantly alter the host’s osteoimmunological response. In diabetic patients, the persistent hyperglycemic state creates a pro-inflammatory microenvironment that prevents the crucial transition from M1 to M2 macrophage polarization, leading to chronic inflammation rather than bone formation. Similarly, nicotine-induced vasoconstriction in smokers and the inhibited bone remodeling caused by antiresorptive treatments create biological “barrier” that most standard Ti surfaces cannot yet overcome. Bridging this gap requires the development of “instructive” surfaces specifically engineered to modulate these compromised immune environments and restore the balance of osseointegration. The following section explains different aspects of why translating the lab findings and discoveries to clinical solutions is not an easy task (Tuikampee et al., 2024).

### 5.1 Discrepancy between *in vitro* and *in vivo* findings

One of the primary challenges facing novel surface modifications is the discrepancy between promising *in vitro* results and definitive *in vivo* or clinical findings. Innovations such as bioactive surfaces, stem cell-incorporated scaffolds, and specialized drug-eluting implants show great potential in laboratory and preclinical models. However, reviewers often concerned that superiority claims based on enhanced hydrophilicity or nanoscale features require consistent clinical corroboration and standardized metrology across different implant platforms before widespread approval (Alves Pereira et al., 2025), (Gulati et al., 2023).

Currently, most advanced surface modifications designed to promote antimicrobial effects are primarily supported by laboratory settings (*in vitro* and animal studies), with only a few proceedings to robust clinical research. The major limitations in preclinical models that slow down clinical translation include the failure of these models to assess outcomes reliably in the long term and accurately reproduce masticatory loading conditions experienced in the oral cavity. Furthermore, the lack of standardization regarding the experimental setup, cell types, animal models, and measurement parameters across studies hinders the ability to make direct comparisons, clouding the true translational viability of many innovative surfaces (Alves Pereira et al., 2025; Gulati et al., 2023; Zhang et al., 2021; Tahsin, 2025; Jia et al., 2025; James et al., 2025; Fiorillo, 2025).

### 5.2 Stability and durability

For dual-purpose coatings, maintaining long-term stability and durability within the dynamic and chemically aggressive oral environment is important. A critical technical failure observed historically is the delamination of surface coatings under physiological stress. For example, plasma spray coating of hydroxyapatite eventually became obsolete due to coating delamination, which was associated with severe marginal bone resorption. Also, materials like calcium phosphate can promote osteoblast adhesion, however can degrade over time (Tahsin, 2025; James et al., 2025).

Modern thin film coatings like layer-by-layer coatings (LbL) used for drug delivery on implants are very fragile. As they can be easily damaged and worn by mechanical forces at two stages. The first stage is during surgery caused by friction from placing the implant in the bone which then breaks the coating. The second stage is during the use, due to constant forces from chewing, grinding, brushing or cleaning the teeth, thus breaking the coating and causing the drugs to be released too soon. So, its highly challenging to design a coating that is strong enough to survive the surgery and the long term use (Alves Pereira et al., 2025; Gulati et al., 2023; Zhang et al., 2021; Tahsin, 2025; Jia et al., 2025; James et al., 2025; Fiorillo, 2025).

### 5.3 Regulatory and long-term safety concerns

Bringing new implant surface technologies into real clinical use is not easy, specifically when drug release approaches or advanced coatings are used. There is no long-term clinical data regarding these new materials, so there is no realistic indication about the long-term safety for such materials. Furthermore, the nano sized particles such as silver nanoparticles can cause cell damage, trigger inflammation or even lead to bacterial resistance (Kakde et al., 2024). Also, Ti itself can wear and corrode releasing ions that can activate the immune system. Another important aspect is the sterilization process of the implant after manufacturing, which can surprisingly change the surface chemistry or structure of the coating, thus creating more implant related problems before its even used (Intravaia et al., 2023; Wei et al., 2022).

### 5.4 Antimicrobial resistance and biofilm resistance

Balancing bone bonding and infection control on implant surface is not an easy task. As rough textures help bone grow into the implant but at the same time can lead to bacterial adhesion and biofilm formation. The development of antimicrobial coating emerged as a solution for such problems; however, they bring other issues as their effect is usually time limited. Additionally, bacteria can develop resistance to the released drug, even smart nano materials that disrupt bacteria can act as a shield for the living ones beneath. Also, adding a strong bactericidal component can unintentionally disturb normal cell behavior and soft tissue integration. So achieving such balance is very critical thus difficult to achieve (Alves Pereira et al., 2025; James et al., 2025; Fiorillo, 2025; Kakde et al., 2024).

### 5.5 Mechanical mismatch and stress shielding

One of the main goals of biomaterial research is to design materials whose mechanical properties match those with human bones. This is needed to not overload the surrounding tissues. Usually the current implantable materials are way stiffer than the bone, causing stress shielding and then leading to bone resorption (Ramaglia Amadasi et al., 2025). Furthermore, most data on mechanical properties and performance still comes from lab tests, which cannot accurately reproduce complex dental chewing forces. For that, well designed animal/clinical models are needed to assess the implant performance under functional loading. Other materials such as zirconia Zr, suffer from brittleness in high load region, shedding lights on other options such as Ti- Zr alloys which provides improved strength and toughness (Alves Pereira et al., 2025).

## 6 Outlooks

The path to develop Ti dental implants that can strongly bind with bone and provide antibacterial protection depends on combining both cutting edge manufacturing with smart biological design. As Ti implants with dual functionality rely on integrated platforms that allow highly precise surface design, responsive interactions with surrounding microenvironment and reliable large scale production so that whatever material produced in lab, can be used safely clinically (Zhang et al., 2021). Overall, conventional methods (grit blasting, etching) are simple and clinically validated but lack antibacterial specificity. Biological coatings (peptides, growth factors) offer dual functionality but face stability and cost challenges. Drug/ion release systems provide strong antibacterial effects but raise concerns about resistance and toxicity. Smart coatings are conceptually attractive yet remain largely experimental. These unresolved controversies underscore the need for comparative clinical trials rather than isolated *in vitro* or small animal studies.

### 6.1 Integrating 3D printing with surface functionalization

Dental implantology field is rapidly changing, the credit is for the advancements in digital technologies specifically 3D printing. Techniques like selective laser melting and electron beam melting are necessary as they allow us to create custom made implants tailored specifically to each patient (Alqutaibi et al., 2024).

Such technologies open many possibilities to overcome current challenges. For example, 3D printing can produce Ti implants with complex porous structures that have the natural flexibility of the jawbone. This porous design acts to increase the surface roughness, which helps bone cells to attach and grow while improving the nutrient flow, aiding the healing and reducing stress shielding. For example, implants can be designed to have a strong dense core with porous outer layer to optimize both durability and bone integration. Furthermore, 3D printing can create implants with a shape that perfectly match the patient tooth root, which aids in reducing the complexity of surgery and speeding up recovery (Zhang et al., 2021; Li B. et al., 2025; James et al., 2025).

One of the most significant advancements is when 3D printing is combined with nanoscale surface engineering. Additive manufacturing allows the integration of multifunctional coatings that can respond to biological stimuli. For example, anti-bacterial agents like silver, zinc, copper, Ti nanotubes or biopolymers can be incorporated or coated into Ti surface to enhance its anti-microbial properties and bioactivity. Such approach and using this type of technology offers a promise to generate smarter and more effective dental implants (Alqutaibi et al., 2024; James et al., 2025; Chahine et al., 2008; Cheng et al., 2014).

### 6.2 Immune instructive surfaces

The future of dental implants goes beyond just being biocompatible, however they are expected to actively guide the body’s healing response. Which means designing implants that facilitate tissue regeneration instead of inflammation. This is mainly achieved by influencing macrophages to polarize the M2 phenotype thus preventing inflammation and supporting bone regeneration and growth. By adding molecules like IL-4 or releasing anti-inflammatory drugs like dexamethasone, implants can reduce the fibrous capsule formation and enhance osteogenesis (Li B. et al., 2025; Chopra et al., 2024).

Looking forward, the main goal is to develop smart implants that can respond to their surrounding microenvironment. By detecting changes like early presence of bacteria or inflammation and release antibiotics or growth factors only on demand. This approach would provide ongoing protection and support without risk of releasing drug in high concentrations all at once, thus making the process safe and more effective.

### 6.3 Interdisciplinary material biology interfaces

To create a next-generation implant, close teamwork between materials scientists, bioengineers, and clinicians is needed. The aim is to shift toward materials that actively join in the body’s natural healing process, not just act as a replacement for missing tissue. For that, a multiscale design that connects tiny atomic level tweaks with real world clinical results is needed. When biomimicry is applied, smart systems like using dragonfly wings to develop surfaces, combined with nanotechnology, material science, and microbiology to kill biofilm, enhance mechanical properties and support healthy bone formation (James et al., 2025).

### 6.4 Scalable manufacturing and personalized medicine

Although patient specific implants have a lot of potential, their clinical success depends on scalable and reasonably price manufacturing (Zhang et al., 2021; James et al., 2025). Resolving issues like high costs that currently restrict use and residual stress in 3D printing. Simple and inexpensive approaches like EA, avoid costly multi step procedures and produce bioinspired nano-scale textures like spikes on rough Ti surfaces, are promising solutions. With surface engineering providing on demand antibacterial/regenerative response, the goal is to develop smart long term implants where 3D printing create custom bone friendly material that can release drugs on demand (Chopra et al., 2024).

### 6.5 Limitations of current evidence and future research needs

Most current evidence on Ti surface modifications derives from *in vitro* assays or short-term small animal models. While these provide mechanistic insights, they oversimplify the complex oral environment, lack patient heterogeneity, and rarely capture long-term remodeling or biofilm succession. Translation to clinical practice therefore remains limited. Key gaps include understanding how immune–osteogenic balance is maintained, how soft tissue seals are stabilized, and how biofilm resilience interacts with osteogenesis under patient risk factors. Future studies should address specific questions: What elastic modulus minimizes crestal bone loss under realistic loading? How do defined nano-topographies modulate macrophage polarization over months? Which surface chemistry enhances epithelial hemidesmosome formation? Addressing these questions through standardized *in vitro* models, large animal validation, and risk-stratified clinical trials will be essential for moving beyond proof-of-concept toward durable clinical success.

## 7 Conclusion

Ti and its alloys are the backbone of dental implantology, however, their bio-inert properties and susceptibility to microbial colonization, continue to be a major clinical challenge. This review emphasized that the future of dental implant success will be to move from passive to smart multifunctional interfaces. By combining cutting edge nano topographies with bioactive layers and ion doping, current surface engineering technologies can potentially achieve both rapid osteointegration and effective antibacterial protection. More specifically, the modulation of the osteoimmunological microenvironment to promote M2 macrophage polarization has been identified as a promising approach to overcome hypersensitivity and pain. Although new materials such as Zirconia and PEEK have emerged as aesthetic and biomechanical alternatives, the further development of Ti surface modification technology is currently the most promising approach to achieve long term infection free success.
